# Prader-Willi syndrome patient with atypical phenotypes caused by mosaic deletion in the paternal 15q11-q13 region: a case report

**DOI:** 10.1186/s13052-022-01398-0

**Published:** 2022-12-29

**Authors:** Jinying Wu, Meifang Lei, Xuetao Wang, Nan Liu, Xiaowei Xu, Chunyu Gu, Yuping Yu, Wei Liu

**Affiliations:** 1 Tianjin Pediatric Research Institute, Tianjin Children’s Hospital (Children’s Hospital of Tianjin University), Tianjin, 300134 China; 2 Tianjin Key Laboratory of Birth Defects for Prevention and Treatment, Tianjin, 300134 China; 3 Department of Neurology, Tianjin Children’s Hospital (Children’s Hospital of Tianjin University), Tianjin, 300134 China; 4 Graduate College of Tianjin Medical University, Tianjin, 300070 China; 5 Tianjin Children’s Hospital (Children’s Hospital of Tianjin University), Tianjin, 300134 China

**Keywords:** Atypical Prader-Willi syndrome, Imprinting defect, Mosaicism, Multi-technology combined diagnosis, Case report

## Abstract

**Background:**

Prader-Willi syndrome (PWS) is a multisystemic complex genetic disorder caused by the loss of paternally expressed genes in the human chromosome region 15q11.2-q13. It is characterized by severe hypotonia and feeding difficulties in early infancy, followed in later infancy or early childhood by excessive eating and gradual development of morbid obesity. Motor milestones and language development are delayed and most patients have intellectual disability.

**Case presentation:**

Here we describe a rare PWS case caused by mosaic imprinting defect in the region 15q11.2-q13 of paternal origin. The proband was a male child with a clinical presentation of global developmental delay and hypotonia with specific facial features. Karyotype of the child was noted as mosaic: 45XY,der(15)?t(15;21),-21[26]/46,XY[24]. Whole-exome sequencing (WES) identified a deletion of 22.7 Mb in size at chr15q11.2q21.1 region and a deletion of 2.1 Mb in size at chr21q22.3 region. The Methylation-specific multiplex ligation-dependent probe amplification(MS-MLPA) of the 15q11.2-q13 region showed that the loading ratio of methylated alleles was 70% and that of unmethylated alleles was 30%(50% normal), which confirmed that the loss of mosaic imprinted defects in the paternal allele led to the diagnosis of PWS.

**Conclusions:**

We propose that complete clinical criteria for PWS should not be considered sensitive in diagnosing partial atypical PWS due to mosaic imprinting defects. In contrast, clinical suspicion based on less restrictive criteria followed by multiple techniques is a more powerful approach.

**Supplementary Information:**

The online version contains supplementary material available at 10.1186/s13052-022-01398-0.

## Background

Prader-Willi syndrome (PWS) is a multisystemic complex genetic disorder caused by functional defects in the paternally imprinted genes in the 15q11.2-q13 region [3]. Clinical manifestations change with age with hypotonia and a poor suck resulting in failure to thrive during infancy. As the individual ages, other features such as short stature, food seeking with excessive weight gain, developmental delay, cognitive disability and behavioral problems become evident [4, 5]. PWS was first described and named by Prader and Willi in 1956 [6]. Besides, it was the first recognized disorder related to genomic imprinting in humans [7, 8]. PWS patients have different clinical characteristics in different growth and development stages, and the prevalence in different populations is about 1/10000 ~ 1/30000 [4, 9]. The criteria for clinical diagnosis were established by consensus in 1993 [10]. These clinical criteria were later modified to help determine who needed further diagnostic testing at different ages . The genetic mechanisms that cause PWS include: 1) paternal 15q11-q13 deletion，about 65% ~ 75%; 2) maternal uniparental disomy (UPD) 15，about 20% ~ 30%; 3) imprinting center microdeletions and variants, about 1% ~ 3% [3, 11]. A multisite cohort study of Molecular genetic classification in PWS summarized genetic data from 510 individuals with PWS and 303 (60%) had the 15q11-q13 deletion; 185 (36%) with UPD15 and 22 (4%) with imprinting defects [12]. Subsequently, definitive molecular genetic tests can be used for the laboratory diagnosis of PWS. There are a number of molecular genetic approaches to confirm PWS, the most common is DNA-based methylation testing. MS-MLPA is the most widely adopted analysis for diagnosing PWS, which can not only diagnose PWS, but also reveal its underlying molecular mechanism [13, 14]. Of note, mosaics in PWS appear to be very rare, especially PWS reports with missing mosaics in the paternal 15q11-q13 region. It is not clear whether the rarity of PWS caused by mosaic imprinting defect is due to the fact that low-to-medium level mosaicism may not be clinically apparent. Here we report a rare case of PWS caused by mosaic deletion in the region 15q11.2-q13 of paternal origin, which was diagnosed by karyotype analysis, WES, and MS-MLPA, despite its atypical clinical features.

### Case presentation

A 10-month-old boy was admitted to the hospital because of global developmental delay. His weight, body length and head circumference were 8.5 kg (P25), 73 cm (P50) and 43 cm (P3) respectively. He cannot roll over or sit alone and his deciduous teeth have not yet erupted. Besides, he could only make the monosyllabic “ba, ma” sounds unconsciously. Physical examination found the patient’s characteristic facies, including almond eyes, short jaw, thin upper lip, turned down corners of the mouth and prominent forehead. Moreover, muscular hypotonia in the extremities and simian crease were found.

The patient was the first child of healthy non-consanguineous parents. At the time of the birth, the mother was 32 years old and the father 35. No family history of developmental delay or intellectual disabilities was reported, nor any family history of genetic syndrome or congenital disease. The mother did not smoke, consume either alcohol or drugs during pregnancy, and did not report decreased in fetal movements. At 38 weeks of gestation, the patient was delivered by cesarean section due to abnormal fetal heartbeat, intrauterine hypoxia and low amniotic fluid. The birth weight was 2.6 kg (P5) and length 47 cm (P5-P15). Born with muscular hypotonia, no abnormalities in sucking power, no special feeding required, no abnormal genital development was found. Because of weak cry, poor spirit and neonatal jaundice, he was transferred to the neonatal department for treatment immediately. During the hospitalization, he was fed with normal milk powder and had blood in stool occasionally. He was discharged from the hospital at 14 days and his weight was 2.55 kg (<P1). After discharging from hospital, he underwent breastfeeding. While, the blood was still occasionally present in the stool. And at the age of 1 month, the weight has no gaining compared with his discharging. Unsatisfied with his weight, the parents took him to a gastroenterologist for treatment. Then the weight began to gain and blood in stool disappears after the feeding mode was switched to deep hydrolyzed protein milk powder feeding, consider milk protein and breast milk allergy. Detailed asking for the patient’s developmental milestones, we found the patient weighed 4.5 kg (<P1) and length 59 cm (P5-P15) at the age of 3 months. He could not raise his head in prone position until 5 months old. When 9 months old, he weighed 7.6 kg (P5-P15) and length 69 cm (P5-P15), could not sit, roll over or grab objects. He could only babble and pronounce the lip sound “poof”.

## Methods

### Chromosome karyotype analysis

Metaphase chromosome preparations were obtained from PHA-stimulated lymphocyte cultures from the patient according to standard procedures. Conventional GTG-banding was performed at a 400–600 band level according to standard protocols.

### Whole exome sequencing and analysis

All steps of whole-exome sequencing (WES) were performed by Tianjin Kingmed Center for Clinical Laboratory (Tianjin, China), including genomic DNA extraction, exome library preparation, exome capture, sequencing, and data analysis.

### Methylation-specific multiplex ligation-dependent probe amplification(MS-MLPA)

MS-MLPA was performed on the 15q11.2-q13 region using SALSA MLPA Probemix ME028-B2 PWS/AS kit according to the manufacturer’s guideline.

## Results

**Fig. 1:**
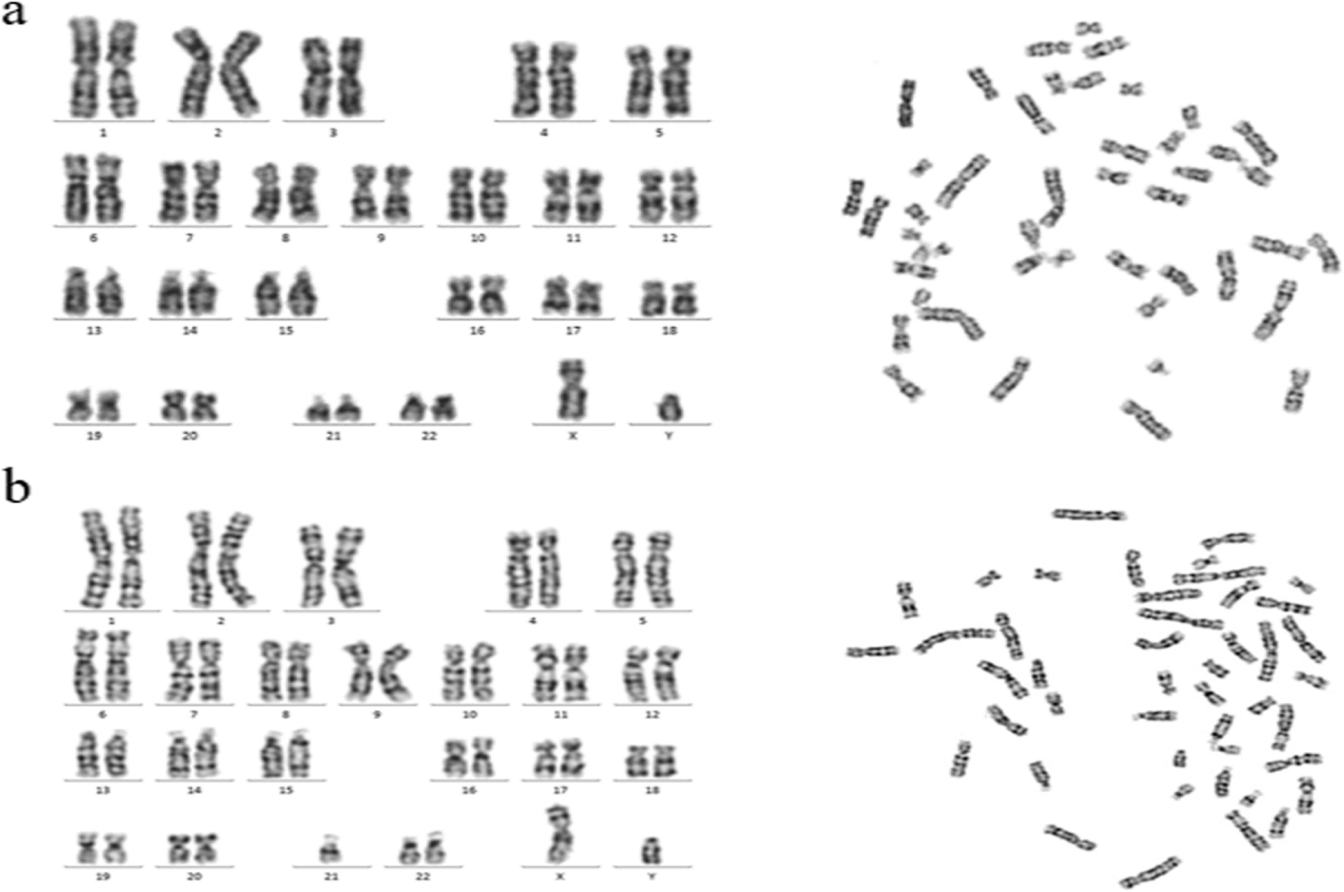
Chromosome karyotype analysis of peripheral blood lymphocytes of children 1a:G-banding in metaphase of peripheral blood lymphocyte division showed chromosome karyotype 46,XY1b:G-banding in metaphase of peripheral blood lymphocyte division showed chromosome karyotype 45XY,der(15)?t(15;21),-21 [1]/46,XY [2])

**Fig. 2:**
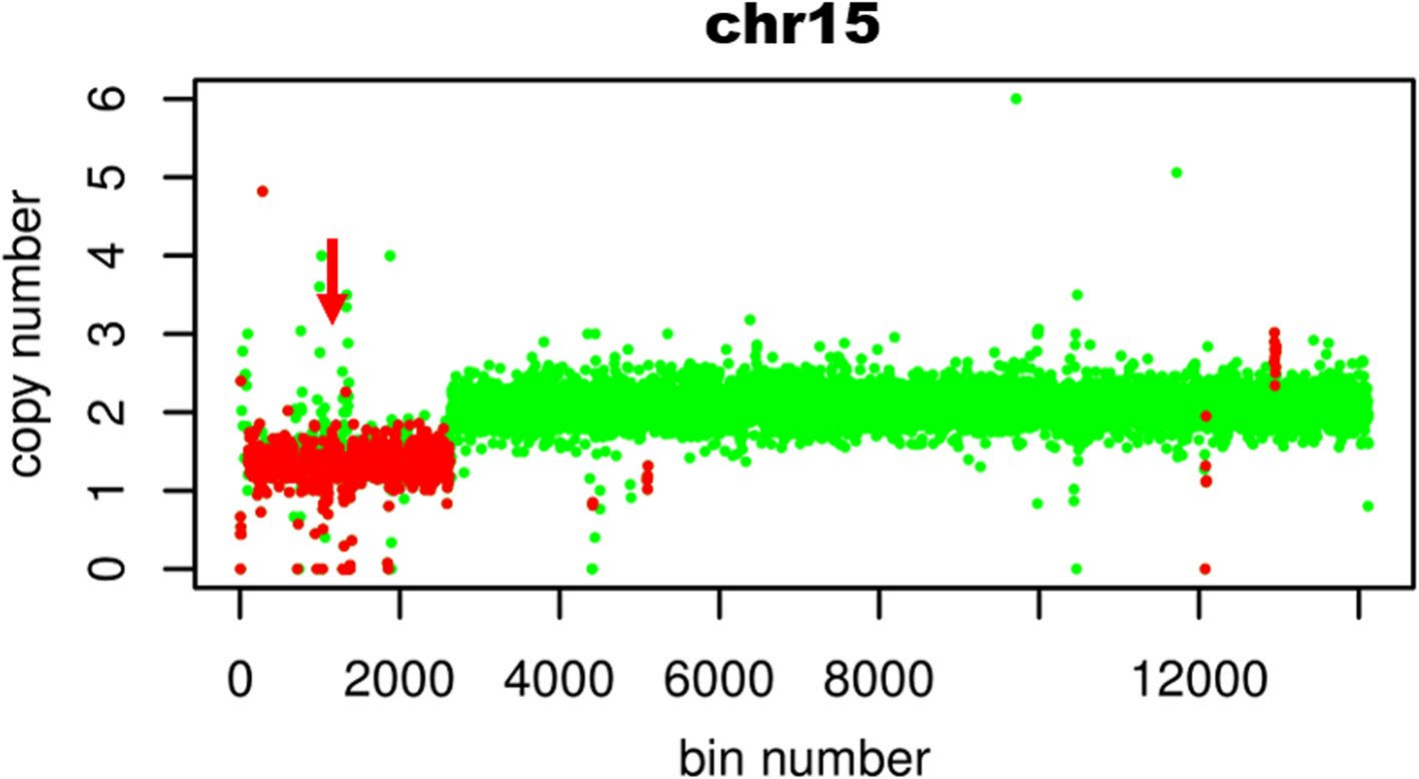
Whole exome sequencing results (There is a fragment deletion on chromosome 15 in the child, and the red arrow shows the deletion region)

**Fig. 3:**
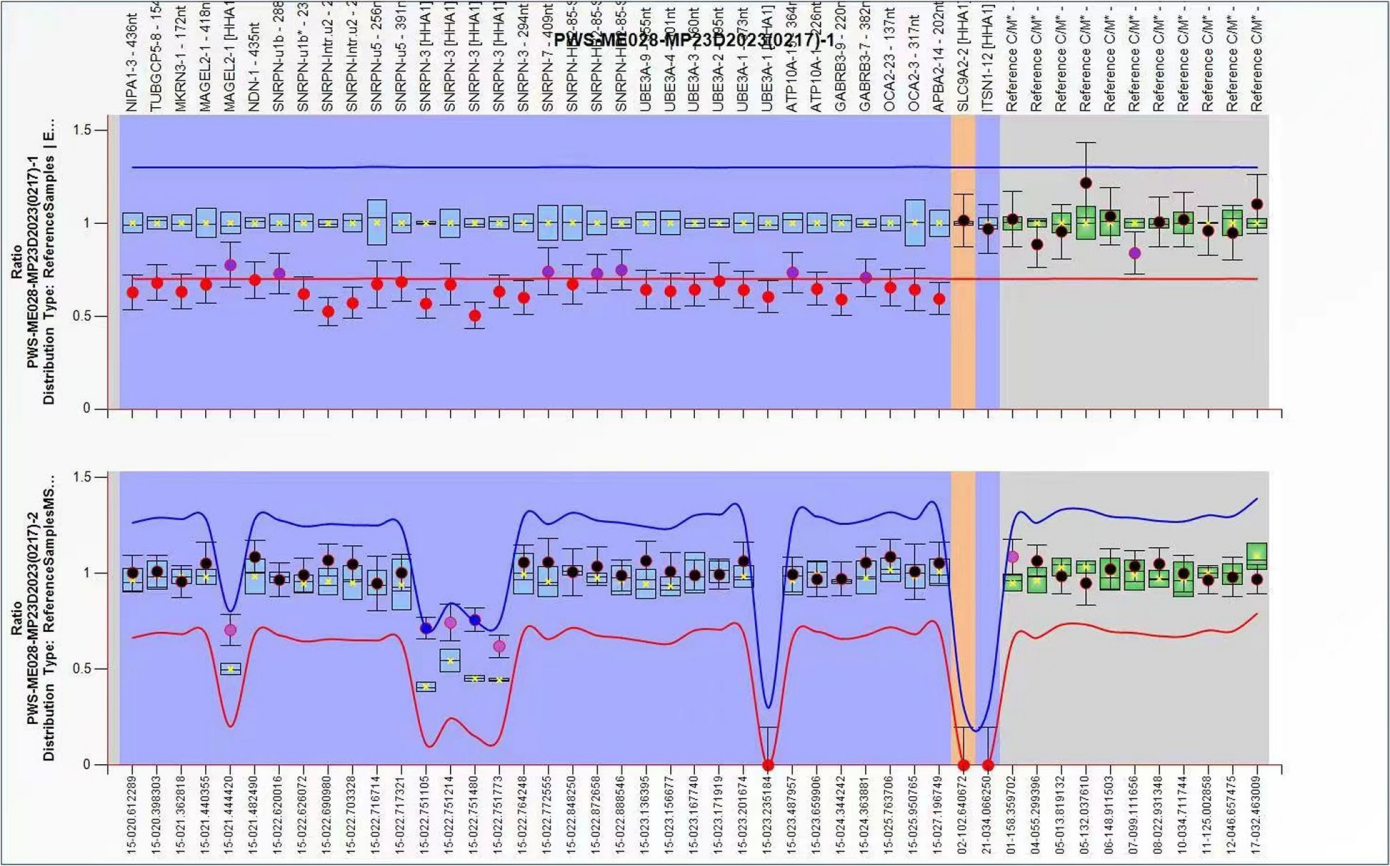
15q11.2-q13 region MLPA analysis results

Cytogenetic analysis revealed a mosaic karyotype (45XY,der(15)?t(15;21),-21 [1]/46,XY [2]) in the proband (see Fig. 1). The changes of chromosomes 15 and 21 need to be identified in combination with genetic results. The chromosomal karyotypes of the parents of the child were normal (see Supplementary Fig. 1 and Supplementary Fig. 2). WES Results showed a deletion of 22.7 Mb in the chr15q11.2q21.1(22833515–45,564,992) region(see Fig. 2) and chr21q22.3 (45817620–47,953,570) region has a deletion of 2.1 Mb(see Supplementary Fig. 3) with a copy number of about 1.5. At present, most of the deletions in the 21q22.3 region coexist with other chromosomal abnormalities and vary greatly in phenotype, ranging from mild to severe, even in patients carrying the same deletion region [15]. So it is difficult to determine the role played by deletions in this region. In our case, it may not work or there may be some synergistic effects that need to be further investigated. The chr15q11.2q21.1 region covers a large number of functional genes associated with diseases including PWS/Angel syndrome, which can explain part of the clinical conditions, and MS-MLPA is recommended for further confirmation. MS-MLPA results showed the copy number of the 15q11-q13 region of the examined sample ranged between 0.5–0.7 (normal value 0.7–1.3), indicating the presence of gene deletion mutation in the patient. Methylation analysis showed a methylation level of approximately 70% (normal value 50%)(see Fig. 3), indicating partial deletion of the methylated paternal allele, due to a mosaic deletion of the paternal allele, consistent with the diagnosis of PWS.

## Discussion and conclusions

Mosaic in PWS appears to be very rare and there are few reports. Izumi et al. [16] reported two infants with PWS caused by mosaic maternal UPD15. Patient 1 has mosaic uniparental isodisomy of the entire chromosome 15, and Patient 2 has mosaic uniparental mixed iso/heterodisomy 15. Wey et al. [17] reported a young woman with an almost typical expression of the PWS due to a mosaic imprinting defect. Huang et al. [18] reported a mosaic de novo *SNRPN* gene variant in a 10-year-old girl who presented with phenotypes that are consistent with the clinical diagnosis of PWS. We know of only two cases of mosaicism of the proximal 15q deletion [19, 20]. On one hand, this may be because that patients with a high proportion of normal cells have milder features, which result in missed diagnosis. On the other hand, it is due to the widespread use of non-quantitative assays to determine methylation status. Our case is interesting mainly because it reports a rare case of PWS caused by a mosaic deletion in the 15q11.2-q13 region of paternal origin. It can enrich the clinical understanding of the atypical clinical phenotype of PWS patients caused by chimerism deletion, help clinical early diagnosis and intervention of the disease, so as to improve the quality of life of patients.

The clinical manifestations of PWS are complex and diverse, covering growth, development, metabolism and other aspects in the life process. Patients in different age groups have different manifestations. For example, one of the typical symptoms in patients, severe obesity due to overeating if uncontrolled, usually occurs between 1 and 6 years of age, so differential diagnosis should be made according to the clinical characteristics of the corresponding age group [3, 7, 21, 22]. In addition, PWS has clinical overlap with other disorders, particularly those with other genetic variants or chromosomal imbalances but with a partially similar clinical presentation to PWS. Sometimes they are referred to as Prader-Willi syndrome-like (PWS-like) [23, 24]. The mosaic of PWS makes phenotypic prediction more difficult. This is not difficult to find by reviewing the clinical phenotypes of previous mosaic deletion cases. Boyd et al. [19] reported a female with clinical features typical of PWS. The karyotype from blood lymphocytes is written as follows: 45,XX, der(1)t(1;15)(p36.3;q12)[50] /46,XX,der(1)t(1;15)(p36.3;q12),+idic(15)(q12)[50]. The methylation studies of exon 1 of SNRPN were normal. The distribution of the deleted cell line appears sufficient to induce the classic PWS symptoms expressed in the individual. Anderlid et al. [20] reported an atypical male patient who had no hypotonia or feeding problems during infancy. There was no formal cognitive assessment during childhood or adolescence, and no apparent behavioral manifestations. At the age of 37, PWS was diagnosed after a visit for nightly sweats and a normocytic normochromic anemia. Our patient had a history of intrauterine hypoxia, a weak cry at birth, and poor spirit. Although no special feeding was required, there was no weight gain for the first month after birth. The patient’s weight, height and head circumference at each stage of growth were below or equal to the average growth standard for same age and sex [2]. The patient’s birth weight was in the 50th centile of the PWS neonatal percentiles may be a further feature supporting the suspicion of neonatal PWS [15]. He is currently 10 months old, unable to roll over and sit alone, and his deciduous teeth have not erupted. Examination of hypotonia, simian crease, exceptional facial features. Our patient did not show the typical clinical features of PWS, partly because the mosaic deletion rescued part of the clinical phenotype, and partly because the patient was still young and some clinical manifestations were not yet apparent. A retrospective study [1] of 90 patients with PWS confirmed by genetic testing found that 15 of the 90 molecularly diagnosed patients did not meet the clinical diagnostic criteria, suggesting that the existing clinical diagnostic criteria may be too exclusionary and suggesting that the clinical diagnostic criteria should be modified to raise diagnostic suspicion at a lower threshold to ensure that all appropriate individuals are tested. This is consistent with the cases we reported and emphasizes the important role of diagnostic tests in confirming the diagnosis of clinically atypical cases.

At present, there is no clear conclusion on the relationship between the proportion of chimerism and the specific phenotypic characteristics of PWS patients. The phenotype might also vary according to the degree of mosaicism in different tissues. A study [25] reported four cases with chimeric deletions of chromosome 15, either with atypical features of PWS or with typical features of PWS, but with undetectable deletions in cytogenetics. Detection using fluorescent in situ hybridization (four commercially available probes) revealed peripheral blood leukocyte deletions ranging from 14 to 60%. The authors suggest that the percentage of cells missing in other tissues may be high in this case. Without knowing the proportion of missing cells in other tissue types and determining more accurately the size of the missing cells, it is not possible to determine the association between genotype and phenotype. To clarify the relationship between clinical symptom typicality and the level of methylated cells, it is necessary to investigate the tissues directly involved in the disease and to quantitatively analyze patients with atypical PWS, and additional studies are needed.

In summary, we report one patient with atypical PWS due to a mosaic deletion in the region 15q11.2-q13 of paternal origin and suggests that the diagnosis of PWS should not be excluded clinically in the case of incomplete PWS phenotypes. The data collected by a combination of the conventional cytogenetic tools, and molecular genetic methods thus enable identification of atypical and complex disease mechanisms. This provides yet another novel insight into the understanding of a mosaicism imprinting disorder.

## Supplementary Information

**Additional file 1:** **Supplementary Fig. 1.** G banding in metaphase of peripheral blood lymphocytes of the father showed chromosome karyotype 46,XY.

**Additional file 2:** **Supplementary Fig. 2.** G banding in metaphase of peripheral blood lymphocytes of the mother showed chromosome karyotype 46,XX.

**Additional file 3:** **Supplementary Fig. 3.** Whole exome sequencing results (There is a fragment deletion on chromosome 21 in the child, and the red arrow shows the deletion region).

## Data Availability

The data that support the findings of this study are available from the corresponding author upon reasonable request.

## Abbreviations

PWS: Prader-Willi Syndrome
WES: Whole-Exome Sequencing
MS-MLPA: Methylation-Specific Multiplex Ligation-Dependent Probe Amplification
UPD: Uniparental Disomy
MS-PCR: Methylation Sensitive Polymerase Chain Reaction
