# Correction: Prader-Willi syndrome patient with atypical phenotypes caused by mosaic deletion in the paternal 15q11-q13 region: a case report

**DOI:** 10.1186/s13052-023-01433-8

**Published:** 2023-03-20

**Authors:** Jinying Wu, Meifang Lei, Xuetao Wang, Nan Liu, Xiaowei Xu, Chunyu Gu, Yuping Yu, Wei Liu

**Affiliations:** 1 Tianjin Pediatric Research Institute, Tianjin Children’s Hospital (Children’s Hospital of Tianjin University), Tianjin, 300134 China; 2 Tianjin Key Laboratory of Birth Defects for Prevention and Treatment, Tianjin, 300134 China; 3 Department of Neurology, Tianjin Children’s Hospital (Children’s Hospital of Tianjin University), Tianjin, 300134 China; 4 Graduate College of Tianjin Medical University, Tianjin, 300070 China; 5 Tianjin Children’s Hospital (Children’s Hospital of Tianjin University), Tianjin, 300134 China

**Correction: Ital J Pediatr 48, 204 (2022)**

**https://doi.org/10.1186/s13052-022-01398-0**

The original article [1] mistakenly mis-numbered a section of citations in the main body; this has since been rectified.
