# Supplementary figures and images for: Prader-Willi syndrome patient with atypical phenotypes caused by mosaic deletion in the paternal 15q11-q13 region: a case report
Source: Ital J Pediatr. 2022 Dec 29;48:204. doi: 10.1186/s13052-022-01398-0 (PMC9798715; doi:10.1186/s13052-022-01398-0)

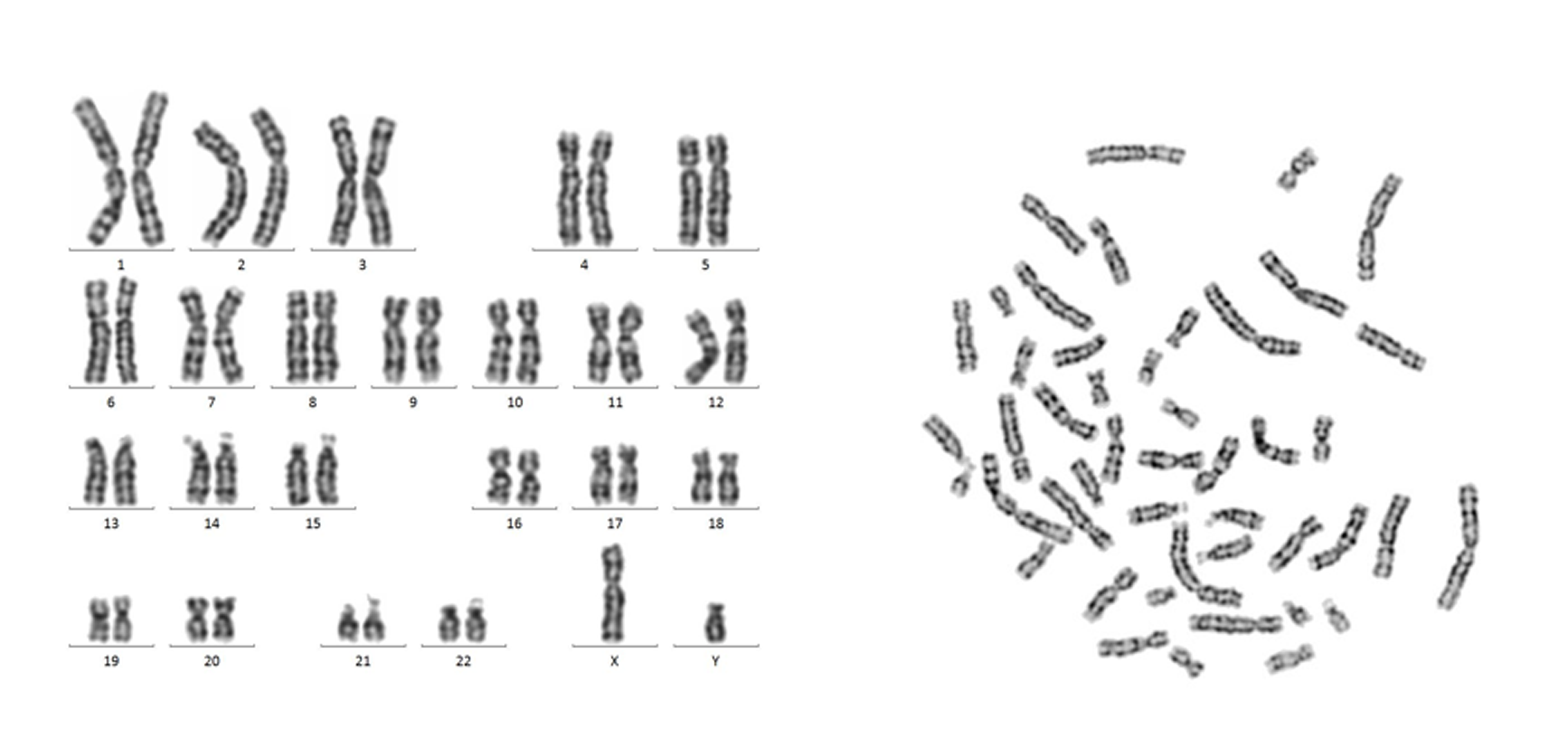

Supplement: Supplementary file 1 — Additional file 1: Supplementary Fig. 1. G banding in metaphase of peripheral blood lymphocytes of the father showed chromosome karyotype 46,XY. [file 13052_2022_1398_MOESM1_ESM.tif]

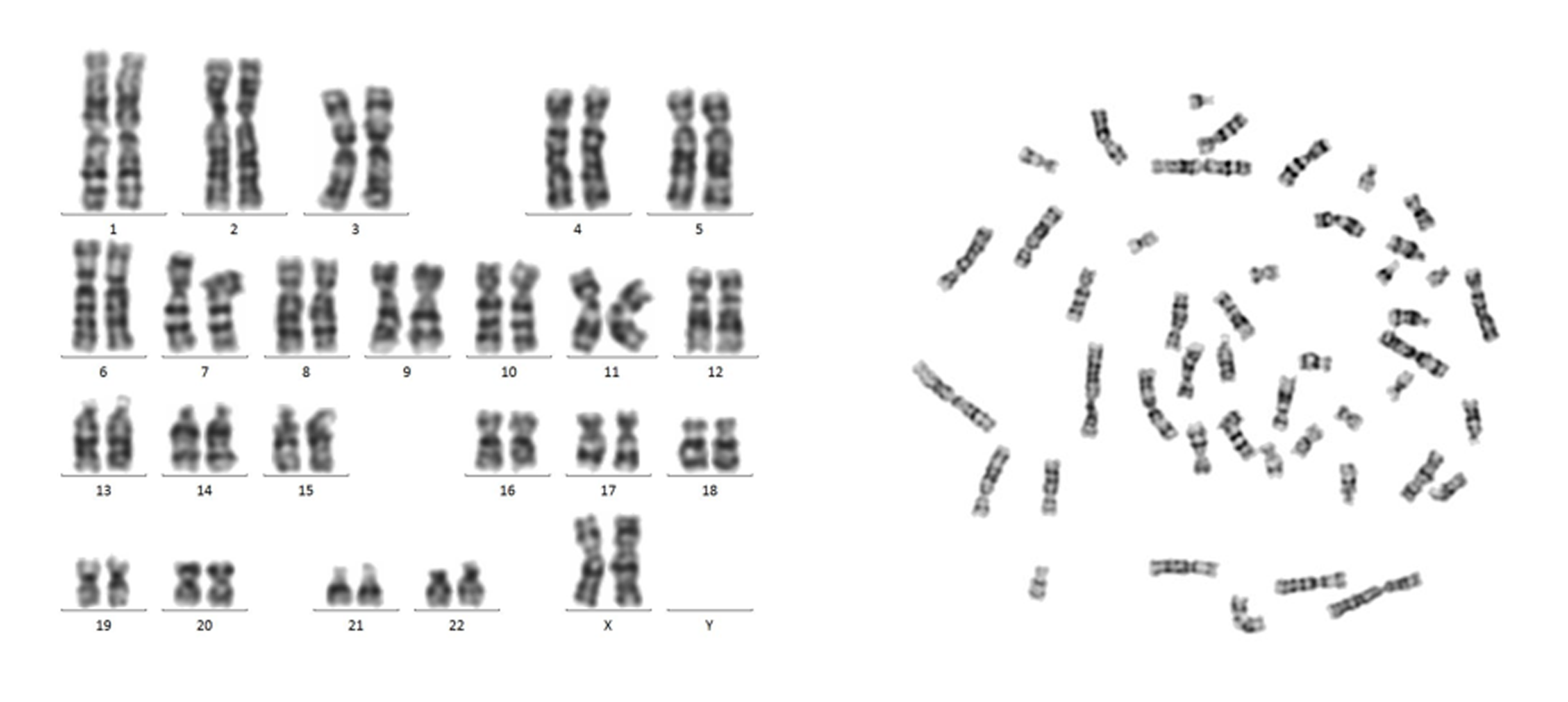

Supplement: Supplementary file 2 — Additional file 2: Supplementary Fig. 2. G banding in metaphase of peripheral blood lymphocytes of the mother showed chromosome karyotype 46,XX. [file 13052_2022_1398_MOESM2_ESM.tif]

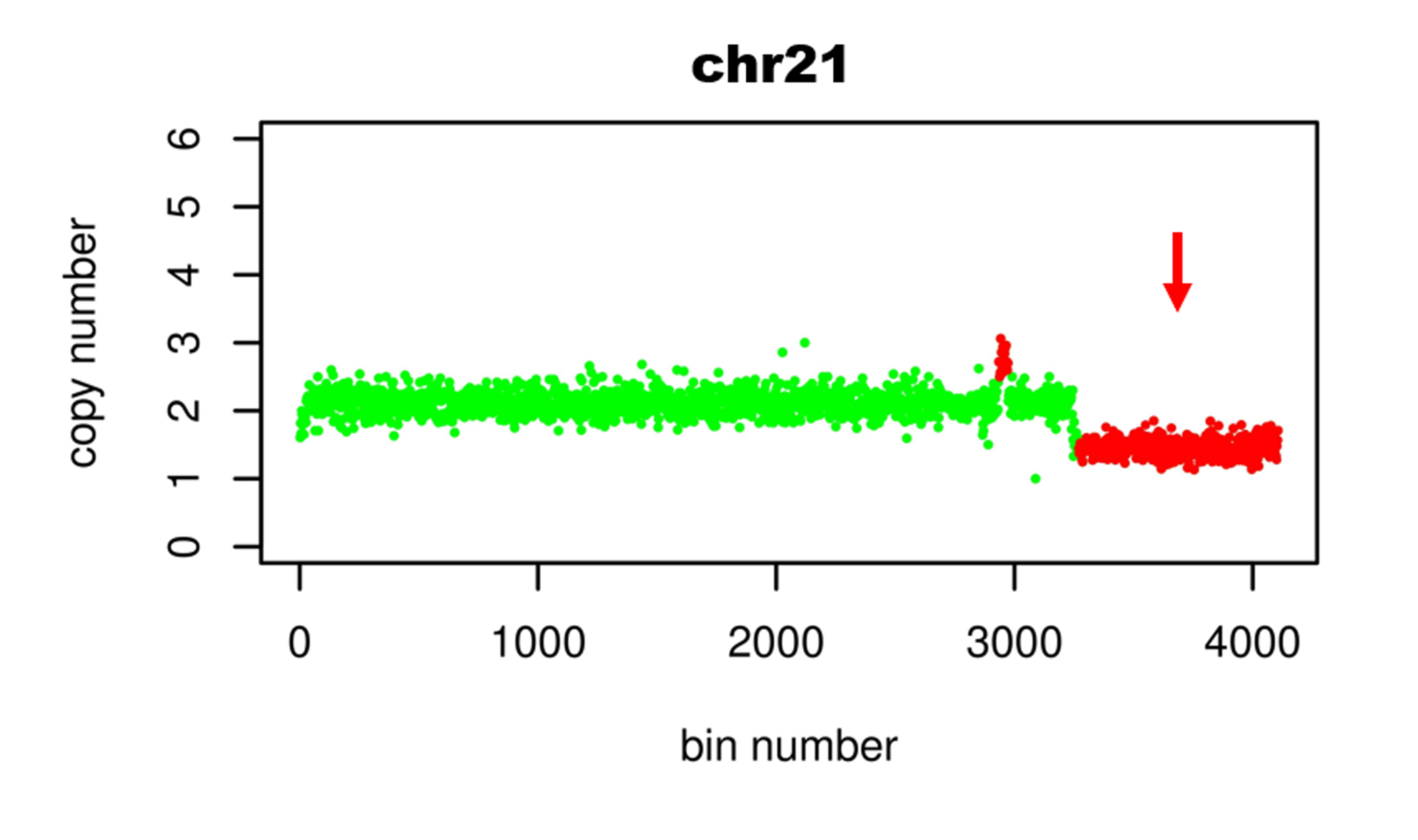

Supplement: Supplementary file 3 — Additional file 3: Supplementary Fig. 3. Whole exome sequencing results (There is a fragment deletion on chromosome 21 in the child, and the red arrow shows the deletion region). [file 13052_2022_1398_MOESM3_ESM.tif]
